# Emerging technologies for detecting antibiotics in aquaculture wastewater: A critical review

**DOI:** 10.1016/j.ese.2025.100572

**Published:** 2025-05-17

**Authors:** Xinyu Chang, Junchi Cui, Guihua Wang, Shujuan Meng, Lingling Chen, Meng Zhang

**Affiliations:** a School of Electronic and Information Engineering, Beihang University, Beijing, 100191, China; b School of Environment, Beijing Normal University, Beijing, 100875, China; c School of Materials Science and Engineering, Beihang University, Beijing, 100191, China; d College of Health Science and Environmental Engineering, Shenzhen Technology University, Shenzhen, 518118, China

**Keywords:** Antibiotics, Analytical methods, Detection, Environmental samples, Wastewater

## Abstract

Antibiotic release and transfer within environmental systems significantly impact ecological stability and human health, posing considerable safety risks. Consequently, accurate and efficient detection methods for antibiotics, particularly within complex environmental matrices, are essential. A growing body of research is focusing on antibiotic pollution in wastewater and other environmental settings. Nevertheless, pervasive matrix interferences in complex water matrices and the inherent limitations of standalone analytical approaches in sensitivity, detection range, and other performance metrics underscore the demand for more robust and versatile detection platforms. Here we show a comprehensive overview and critical assessment of antibiotic contaminants, examining their sources, environmental distribution, and current detection methods. We focus on emerging antibiotic detection technologies, comparing their performance and highlighting suitable application contexts. Furthermore, we discuss fundamental principles and historical advancements in antibiotic detection and analytical methodologies. Finally, we identify significant challenges for antibiotic detection in complex environments, suggest viable strategies for future improvements, and outline promising research directions. This review not only provides essential guidance for advancing environmental antibiotic monitoring but also sheds light on the development of a strategic framework for robust, integrated platforms enabling multiplexed antibiotic monitoring in challenging environmental water matrices.

## Highlights

• This review discusses recent advancements in antibiotic detection and their underlying analytical principles.
• The strengths, limitations, and future potential of emerging antibiotic detection methods are critically assessed.
• Major challenges of antibiotic detection in complex environments are identified, with strategies for future research.

## 1 Introduction

In recent years, antibiotics have become prominent due to exponential growth in their usage, which has established them as a significant category of emerging contaminants. Antibiotics constitute a unique class of substances primarily synthesized by microorganisms, including bacteria, fungi, and higher organisms, during their life activities. The main functions of antibiotics are to combat pathogens, inhibit bacteria, and disrupt the normal development of other living cells, including cancer cells. The primary mechanisms of action include inhibition of bacterial cell wall synthesis, enhancement of bacterial cell membrane permeability, disruption of bacterial protein synthesis, and inhibition of bacterial nucleic acid replication and transcription. Since their discovery and subsequent mass production, antibiotics have emerged as indispensable tools for preserving human health and the health of various animals and plants. The introduction of antibiotics has resulted in groundbreaking changes in fields such as medicine, biological cytology, livestock breeding, agricultural production, and aquaculture. However, with human society's advancement in production and daily life, the use of antibiotics has become widespread and extensive [[1], [2], [3], [4], [5]]. In a study conducted at the University of Oxford, researchers statistically analyzed antibiotic consumption in more than 200 countries over the past two decades [6]. The study revealed a steady global increase in antibiotic consumption, amounting to an approximately 46 % increase. This increase has resulted in large quantities of unconsumed and unmetabolized antibiotics entering surface water, soil, and groundwater through excretion by organisms. These antibiotics ultimately migrate continuously between water bodies and soil, leading to continuous accumulation. The significant presence of antibiotics in aquatic environments has the potential to disrupt the balance of microorganisms in ecosystems [7], induce antibiotic resistance in diverse bacteria [8], pose risks to human health [9], and ultimately contribute to a decline in biodiversity and ecosystem degradation [10,11]. Considering the increasingly severe public health situation, there is a growing and urgent need for efficient and convenient methods to detect antibiotics in water environments.

Traditionally, antibiotic detection relies on methods such as high-performance liquid chromatography (HPLC), mass spectrometry (MS), and the combined approach of liquid chromatography‒mass spectrometry (LC‒MS) [[12], [13], [14]]. Chromatography, in which a liquid is employed as a mobile phase, is the primary tool for detecting antibiotics in water. This method is grounded in the principles of traditional liquid chromatography, incorporates elements of gas chromatography theory, and utilizes equipment such as infusion pumps, separation columns, detectors, and computer control systems for analysis [15,16]. In contrast, MS is a technique that uses electric and magnetic fields to separate moving ions (e.g., charged atoms, molecules, or molecular fragments) based on their mass-to-charge ratios. MS can be used to measure the precise mass of these ions and to determine the composition of compounds. LC‒MS combines the separation capability of liquid chromatography with the measurement precision of mass spectrometry [[17], [18], [19], [20]]. This hybrid approach boasts greater analytical power and sensitivity than individual techniques; however, its enhanced capabilities require the use of more intricate technology and equipment, leading to increased detection costs. While these aforementioned methods are considered foundational in antibiotic detection, they are limited by their lengthy analysis times, intricate procedures, high costs, and complex sample preparation methods.

**Fig. 1:**
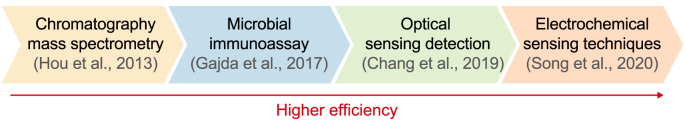
Overview of methods used for antibiotic detection [29,30,95].

Considering the problems facing antibiotic detection, developing novel responsive detection technologies and integrated sensor devices is crucial. In recent years, numerous researchers have introduced various detection and analysis techniques [[21], [22], [23]]. Innovative techniques such as chromatography‒mass spectrometry, optical sensing, and electrochemical sensing have gained prominence in wastewater antibiotic detection [[24], [25], [26]]. In 2008, Xiao and colleagues at Peking University introduced a liquid chromatography‒electrospray tandem mass spectrometry method for analyzing quinolone antibiotics in wastewater. Shortly thereafter, researchers at Zhejiang University devised a liquid chromatography‒fluorescence method coupled with one-step solid-phase extraction to comprehensively detect multiple antibiotics in water [27]. In 2020, scholars at Donghua University proposed an impedance aptasensor based on TiO_2_-g-C_3_N_4_@AuNPs for the ultrasensitive detection of amoxicillin [28]. As shown in Fig. 1, these innovative approaches represent a crucial shift toward more efficient and convenient methodologies in the continuous quest for precise antibiotic detection. Moreover, developing integrated sensor devices enables more accurate detection and identification of antibiotics, providing more effective solutions for monitoring water environments.

This review explores the distribution and sources of common antibiotics in diverse aquatic environments. It also offers an overview of the advancements in techniques for detecting and analyzing antibiotics while elucidating the principles underlying these methods. Furthermore, common antibiotic detection methods are comparatively analyzed, and the strengths of different approaches and their future potential are critically summarized. In the conclusion of this article, emerging trends in the detection of antibiotics in complex environments, including advancements in bioscience, optical sensing, and sensitive materials, are explored. Considering the importance of antibiotic detection in areas such as ecological environments and wastewater management, several potential new methods hold great promise for advancing antibiotic detection technologies. Moreover, the numerous challenges associated with the detection of antibiotics in complex environments are discussed, and potentially feasible strategies and directions for the future development of antibiotic detection are envisioned.

## 2 Antibiotics commonly found in wastewater

Significant amounts of antibiotics enter aquatic environments, primarily through the discharge of domestic sewage [31], medical wastewater [32,33], industrial wastewater [34,35], and wastewater from animal husbandry and aquaculture [[36], [37], [38]]. Among these sources, the supervision and treatment of domestic sewage, as well as medical and industrial wastewater, has been significantly improved by environmental agencies, primarily through establishing sewage treatment plants and medical waste management systems. Antibiotics from domestic sewage and medical wastewater are eventually discharged into wastewater treatment plants. Factories and enterprises must install appropriate sewage treatment equipment to remove pollutants from industrial wastewater, including antibiotic residues [39]. Numerous studies have demonstrated that adsorption and biodegradation, as established methods, are widely employed [40,41]. Furthermore, increased public awareness of antibiotic use in medical contexts has substantially reduced antibiotic residues in domestic sewage and medical wastewater [42]. Conversely, globally, over half of antibiotics are utilized in the production of food animals, with numerous livestock and aquaculture enterprises being the primary consumers of these drugs [43,44]. In large-scale breeding, antibiotics are used to prevent and treat animal infectious diseases and added to feed and drinking water as growth promoters [45]. Owing to their inherent characteristics, antibiotics are challenging to metabolize within organisms. As a result, substantial amounts of antibiotics are released into the environment with feces and other excreta [12]. Next, we analyze each type of wastewater individually.

### 2.1 Domestic sewage

In many countries, antibiotics are unregulated, which allows patients to purchase them independently and use them at home. The most commonly used antibiotics are penicillins and β-lactams [31]. Thus, antibiotics can be directly released into the environment by patients. Drugs that are not fully metabolized by the human body may also enter the sewage treatment system via urine or feces. In addition, some unused or expired drugs may be discarded, contributing to antibiotic contamination in aquatic environments [46]. As public awareness of medical and environmental issues has improved, the abuse of antibiotics has gradually decreased. However, the proper disposal of unused and expired drugs still requires significant attention from governments and international organizations, along with the enhancement of relevant laws and regulations and the implementation of appropriate measures [47].

### 2.2 Medical wastewater

Hospitals and medical activities play a crucial role in maintaining public health. Moreover, the threat posed to the ecosystem by medical wastewater must be considered. Medical wastewater is a primary source of antibiotics entering aquatic environments, soil, crops, and sewage treatment plants [[48], [49], [50]]. The characteristics of medical wastewater vary depending on factors such as geographic region, prescribed antibiotic type, type of medical institution, bed density, ward type, country, and seasonal variations [50]. Reports have indicated that antibiotics such as enrofloxacin, ciprofloxacin, ofloxacin, norfloxacin, sulfamethoxazole, trimethoprim, and metronidazole, as well as their metabolites, are present at high concentrations in hospital wastewater [49,51]. Among the various antibiotic transmission pathways, hospital wastewater collectors and sewage treatment plants play significant roles [52]. For several antibiotics, the removal efficiency of conventional treatment processes in sewage treatment plants is limited [53], necessitating the use of real-time online detection methods.

### 2.3 Industrial wastewater

Industrial wastewater is another significant source of antibiotics in aquatic environments. Notably, the concentration of antibiotics in wastewater from pharmaceutical factories is several orders of magnitude higher than that in domestic wastewater, reaching mg L^−1^ levels [54,55]. Reports have indicated that pharmaceutical wastewater from industrial production is a major contributor to elevated antibiotic residues in water environments in Europe [56] and Vietnam [57]. Similarly, in the Pudong New Area (Shanghai, China), industrial pharmaceutical wastewater is the primary source of antibiotic pollution in surface water [58].

### 2.4 Livestock breeding wastewater

Pig breeding is a common type of domestic livestock breeding. The antibiotics most widely used in the domestic pig breeding industry can be broadly categorized into four groups: sulfonamides, tetracyclines, macrolides, and chloramphenicol [59,60]. Table 1 shows the proportions of antibiotics used in livestock and meat production worldwide in recent years [61]. Domestic pig breeding is mainly individualized and free-range in vast areas of the world, including developing countries such as China, with a low degree of centralization. A previous study indicated that the majority of farmers and rural individuals lack accurate knowledge regarding the purpose of antibiotic use and the usage and dosage of specific antibiotics [[62], [63], [64]]. This disparity has led to many antibiotics in domestic pig manure. Approximately 30–90 % of antibiotics, depending on the type of antibiotic, enter water bodies, sediments, and soil through fecal matter [60,65,66].

**Table 1:** The usage rates of different antibiotics in the livestock industry [61,63].

Penicillins (87.1 %)	Tetracyclines (87.1 %)	Macrolides (77.1 %)	Sulfonamides (70.0 %)	Quinolones (68.6 %)
Benzylpenicillin	Chlortetracycline	Tulathromycin	Sulfachlorpyridazine	Flumequine Miloxacin Nalidixic acid Oxolinic acid Ciprofloxacin Danofloxacin Difloxacin Enrofloxacin Marbofloxacin Norfloxacin Ofloxacin Orbifloxacin
Penethamate hydroxide	Doxycycline	Erythromycin	Sulfadiazine	
Penicillin procaine	Oxytetracycline	Josamycin	Sulfadimerazin	
Mecillinam	Tetracycline	Kitasamycin	Sulfadimethoxine	
Amoxicillin		Spiramycin	Sulfadimidine	
Ampicillin		Tilmicosin	Sulfadoxine	
Amoxicillin-clavulanic		Mirosamycin	Sulfafurazole	
Ticarcillin		Terdecamycin	Sulfaguanidine	
Tobicillin			Sulfamethazine	
Aspoxicillin			Sulfadimethoxazole	
Phenoxymethylpenicillin			Sulfamethoxine	
Phenethicillin			Sulfamonomethoxine	
Penicillin			Sulfaquinoxaline	
Cloxacillin			Trimethoprim	
Dicloxacillin			Sulfonamide	
Nafcillin			Baquiloprim	
Oxacillin			Trimethoprim	

### 2.5 Aquaculture wastewater

The Food and Agriculture Organization defines aquaculture as the cultivation of aquatic organisms, including fish, mollusks, crustaceans, and aquatic plants. The term “aquaculture industry” refers to a set of practices aimed at improving production efficiency. These practices may involve regular feeding, the use of medicines and fertilizers, and other methods to ensure yields. In aquaculture, antibiotics mainly treat common diseases and promote growth and development [67]. Common internationally approved antibiotics for aquaculture include fluoroquinolone, β-lactam, tetracycline, macrolide, and sulfonamide antibiotics. However, despite clear regulations on drug usage, a significant amount of misuse still occurs. Moreover, as aquatic drugs have low utilization rates, aquatic products can absorb only 20 %–30 % of these drugs. The remainder, a significant quantity, is discharged into surrounding waters or sediments, contributing to nonpoint source pollution [67]. High concentrations of antibiotics, including norfloxacin, ofloxacin, amoxicillin, erythromycin, tetracycline, and sulfamethoxazole, are commonly detected in aquaculture wastewater. These antibiotics have been detected in bodies of water across multiple countries (Table 2).

**Table 2:** Antibiotic concentrations in typical water samples.

Country	Category	Concentration (ng mL^−1^)	Reference
Mozambique	Sulfamethoxazole	5.38 × 10^−1^	[68]
Kenya, Nairobi River	Sulfamethoxazole	3.89 × 10^−1^	[69]
Croatia	Sulfamerazine	1.10 × 10^−1^	[56]
Vietnam, Mekong River	Sulfamethazine	1.91 × 10^−1^	[70]
China, Lake Poyang	Sulfadiazine	5.62 × 10^−2^	[71]
China, Huangpu River	Oxytetracycline	4.87 × 10^−2^	[72]
China, surface water in the Pearl River Delta	Doxycycline	3.97 × 10^−2^	[73]
China, water sources of Wuhan	Sulfadiazine	5.36 × 10^−2^	[74]
China, Yellow Sea coastal area	Sulfamethoxazole	2.59 × 10^−1^	[75]
	Streptomycin	3.41 × 10^−2^	
	Tetracyclines	2.34 × 10^−1^	
	Sulfamethoxazole	5.82 × 10^−2^	
	Enrofloxacin	4.99 × 10^−2^	
	Sulfonamides	1.44 × 10^−2^	
	Ciprofloxacin	2.11 × 10^−2^	
	Enrofloxacin	1.35 × 10^−1^	

**Fig. 2:**
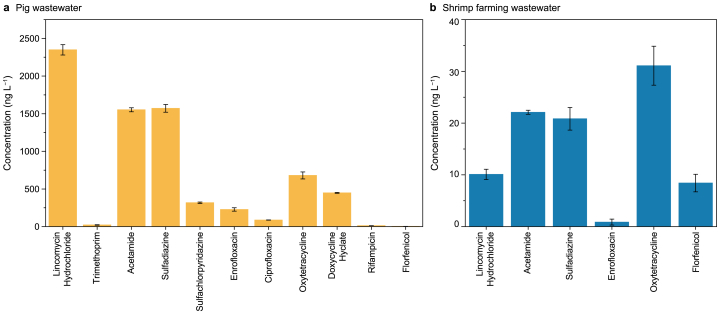
Types and concentrations of antibiotics in pig (**a**) and shrimp farming (**b**) wastewater in Hainan Province, China.

We conducted a field survey to assess the types and concentrations of antibiotics in pig and shrimp wastewater in Hainan Province, China. The concentrations of detected antibiotics were classified and quantified using the HPLC‒MS/MS method (Fig. 2). The results revealed that certain antibiotics in pig wastewater reached concentrations as high as μg L^−1^. The presence of high concentrations of various classes of antibiotics is of considerable concern. The unchecked use and release of antibiotics pose environmental risks and contribute to the development of antibiotic resistance in bacteria. This resistance complicates disease control and has led to the emergence of formidable resistant strains [76]. Therefore, regulating the use of antibiotics in the aquaculture industry is essential for both human health and environmental sustainability.

## 3 Detection of antibiotics in water environments

Antibiotic detection methods are commonly categorized into four groups: chromatography‒mass spectrometry methods [[77], [78], [79], [80], [81]], microbial‒immunoassay techniques [82,83], optical approaches, and electrochemical sensing techniques [[84], [85], [86], [87], [88]]. Previous research has described the principles and applications of HPLC, MS, and LC‒MS. Researchers have developed strategies to detect antibiotics using microorganisms in conjunction with immunoassays. For example, a 2008 study evaluated the growth dynamics of commercial *Pseudomonas aeruginosa* and soil-derived *Pantoea agglomerans* in antibiotic assays based on pH-dependent inhibition [89]. While microbial methods can save time, they may produce false positives or false negatives [83], leading to inaccurate results. Subsequently, new methods that combine the microbial detection of antibiotics with other methods have been proposed [90]. In one study, microorganisms were combined with high-performance liquid chromatography to analyze seven antibiotics, including sulfonamides and quinolones [91]. The antibiotic residue screening test (STAR) method and the 2,3,5-triphenyltetrazolium reduction (TTC) method were compared. The TTC method detected type 5 sulfonamide antibiotics more effectively at concentrations below 50 μg L^−1^ in dairy products than the STAR method. However, the STAR method effectively detects low concentrations of quinolone antibiotics, such as enrofloxacin and ciprofloxacin, in milk. In another study, luminescent-sensing bacteria were used to detect antibiotic concentrations in natural environments [92]. The well-known luciferase gene serves as a reporter gene, causing cells to glow without external light. The bioluminescence intensity is measurable, and cellular damage from antibiotics can trigger various bacterial repair systems. Despite its benefits, such as a short incubation time, this method faces challenges, such as intricate bacterial cultivation. In 2009, the Spanish Network Research Center for Biomaterials and Nanomedicine introduced an immunosensor capable of detecting sulfonamide antibiotics in milk [93]. Based on a microfluidic platform, this immunoassay detects biomolecule adsorption through changes in the waveguide evanescent field, achieving impressive detection limits in both buffer and milk. In the same year, the team introduced a groundbreaking optical biosensor designed around the wavelength principle that is aimed at facilitating the simultaneous screening of multiple analytes, including sulfonamide, quinolone, β-lactam, and tetracycline antibiotics in milk [94]. Using the evanescent wave phenomenon, this sensor effectively detected various antibiotic residues within milk samples through antigen‒antibody binding. The microbial‒immunoassay technique, known for its economical sample requirements, cost effectiveness, and widespread acceptance, possesses commendable attributes. However, certain limitations of this technique must be recognized, such as its intricate operation, reduced sensitivity, strict storage conditions, and limited selectivity.

In recent years, optical and electrochemical methods have been the primary techniques for detecting antibiotics [[95], [96], [97], [98], [99], [100]]. Optical methods have gained traction among scientists because of their straightforward visual results and ease of operation. In 2014, a breakthrough occurred when researchers introduced an enhanced fluorescence system for tetracycline detection that utilizes gold nanoclusters [101]. These nanoclusters act as fluorescence-enhancing agents, building a system around Eu^3+^–tetracycline complex supplemented by gold nanoclusters. This system can detect tetracycline within a linear range of 0.01–5 μM and boasts a detection limit of 4 nM and a signal-to-noise ratio of 3. Moreover, the practicality of this method in actual work (detection of tetracycline in human urine and milk samples) was verified. In addition to fluorescence detection, the colorimetric detection of antibiotics is intuitive. One notable example involves an alkali-etched manganese-based Prussian blue analog known for its superb oxidase-mimicking activity [102], pivotal in a tetracycline colorimetric sensing system. The process involves revealing the sites and surfaces of the analog via etching with NaOH, which notably increases its catalytic ability. Density functional theory is employed in the system to select the best molecularly imprinted polymer (MIP) layers for precise target identification. Its performance was evidenced by the consistent linear relationship with the absorbance at 652 nm for tetracycline concentrations ranging from 0.2 to 200.0 μM, with a detection threshold of 0.07 μM. Moreover, this system adeptly distinguishes specific targets from a variety of interferents. In another innovative project, engineers crafted a detection kit for the onsite identification of oxytetracycline in milk [103]. This ingenious kit simplifies the creation of lateral flow-based aptasensors by attaching tetracycline to a 7 kDa carrier protein and securing it onto a nitrocellulose membrane. Leveraging a 0.125 μM ligand‒gold conjugate, the system can visually identify tetracycline concentrations as low as 5 ng mL^−1^ in milk samples within only 10 min, comfortably meeting the standard threshold of 100 ng mL^−1^. Thus, this kit has emerged as a compelling solution for swift antibiotic detection and is especially crucial for ensuring food safety.

Electrochemistry has recently been used in sensing to detect specific antibiotics concentrations through different indicators, including impedance magnitude, redox peak intensity, and photocurrent response strength. For example, some innovators have crafted sensing probes using carbon quantum dots to amplify electrochemical signals [104]. When Hg ions and these sensing probes are introduced onto an electrode surface, they stimulate electron transfer, thereby increasing the generation of electrochemical signals. This tailored electrode shows excellent electrocatalytic activity toward Hg, with anode and cathode peaks at 0.53 and 0.42 V (vs. saturated calomel electrode [SCE]), respectively. The introduction of amoxicillin disrupts the sensing probe at the interface, suppressing electrochemical signal production. This probe exhibits an ideal linear response from 0 to 100 nM, with the detection threshold reduced to an impressive 15 nM. In a novel approach, researchers introduced a carbon-doped ZnO nanocomposite molecularly imprinted electrochemical sensor tailored for detecting oxytetracycline in milk [105]. When combined with acetylene black, this nanomaterial serves as a reinforcing conductive matrix, enhancing the efficiency of electron migration. Concurrently, based on electropolymerization, the MIP sensor represents a new standard, with a lower detection limit for oxytetracycline of 8.75 pM and a broad linear detection range spanning 0.01–1000.00 nM. Its exemplary selectivity and enduring stability are worth noting.

In conclusion, choosing and implementing techniques for detecting antibiotics in aquatic environments play a pivotal role in maintaining food safety, safeguarding environmental health, and facilitating accurate clinical diagnoses. While numerous methods can detect a variety of antibiotics, the unique chemical structure and properties of each antibiotic often necessitate specific detection approaches. In the following sections, we delve into the specialized detection techniques and nuances associated with several common antibiotics found in wastewater.

### 3.1 Detection of tetracycline antibiotics

In animal husbandry and aquaculture, tetracycline antibiotics are fundamental in antibacterial practices because they display potent efficacy against mycoplasma, chlamydia, spirochetes, and gram-negative and gram-positive bacteria. However, tetracycline resistance genes have been identified in pig farm wastewater, pig manure, and adjacent river systems, indicating substantial residual levels. This accumulation in aquatic environments has raised concerns regarding the environmental impact of these antibiotics [76,106]. The detection of tetracycline antibiotic residues has become imperative.

In recent years, there has been a surge in the development of tetracycline detection methods that leverage fluorescence-based strategies [85,[107], [108], [109], [110], [111], [112]]. Among these advancements, a dual-mode nanosensor that uses reduced carbon dots (r-CDs) to detect tetracycline has been introduced (Supplementary Material Fig. S1a) [113]. This innovative approach operates via a Förster resonance energy transfer mechanism, under which the presence of tetracycline suppresses the fluorescence emitted by r-CDs, enabling highly sensitive detection with a remarkable limit of detection (LOD) of 1.73 nM. Moreover, the color of the r-CDs exhibits an observable shift from colorless to red, providing an alternate detection method visible to the naked eye that achieves an LOD of 0.46 mM. These r-CDs demonstrate specificity and sensitivity in monitoring tetracycline within wastewater, underscoring their practical significance in diverse wastewater monitoring and treatment applications.

#### 3.1.1 Semiconductor material fluorescence-based detection methods

Notably, a pioneering study in 2019 introduced a novel tetracycline fluorescence sensor leveraging molybdenum disulfide nanosheets (MoS_2_ NPs) [114]. The method involves a bottom-up hydrothermal process to synthesize molybdenum disulfide nanoparticles, which exhibit distinctive blue fluorescence at 430 nm. The fluorescence is significantly quenched by adding tetracycline owing to the combined internal filtering effect and electron transfer. This innovation led to the development of an MoS_2_ NP-based fluorescence sensor for tetracycline detection, which demonstrated practicality for examining tetracycline levels in milk powder, milk, and beef samples (with the ability to detect concentrations as low as 0.032 μM and a recovery rate between 88.46 % and 108.62 %).

In a separate breakthrough with exceptionally low detection limits, researchers introduced a zinc oxide tetrapod nanomaterial structure for the fluorescence immunodetection of tetracycline [115]. This novel material has a structure with needle-like branches, which vary in length from hundreds of nanometers to several micrometers, with an average thickness of tens of nanometers (Supplementary Material Fig. S1b). The ample surface area of this structure facilitates efficient bioreceptor immobilization and reactions. These ZnO nanotetrapods exhibit robust photoluminescence (PL) at room temperature, with small surface changes significantly impacting the PL. The PL signal of ZnO decreases with increasing tetracycline concentration, enabling precise measurements from 0.001 to 1.000 μg L^−1^ and an impressive decrease in the detection limit to 1.2 ng L^−1^.

#### 3.1.2 Quantum dot fluorescence-based detection methods

With advancements in material technology, scientific experts have introduced the innovative concept of “quantum dots” as fluorescent probes. Owing to their quantum size, tungsten oxide quantum dots exhibit remarkable optical and electronic properties and have proven to be invaluable fluorescent probes for detecting tetracycline in animal products [116]. In this approach, tungsten disulfide (WS_2_) and hydrogen peroxide (H_2_O_2_) are used as the tungsten source and a sufficient oxidant (Supplementary Material Fig. S1c); additionally, a pot of ethanol is used, and templates, harsh conditions, or hazardous chemicals are not required. Blue luminescent tungsten oxide quantum dots were synthesized using a thermal method. These prepared quantum dots are significantly quenched by tetracycline owing to the collaborative effects of the internal filter effect, fluorescence resonance energy transfer, and photoinduced electron transfer. This method exhibits a linear relationship in detecting tetracycline at concentrations ranging from 0 to 50 μM, with a detection limit of 19 nM. The sensor monitors adulterated milk and milk powder samples, confirming its practical utility. In a separate endeavor, the researchers introduced a portable concept in which a smartphone was integrated with quantum dot fluorescence-based detection. A portable smart sensing platform was developed using silk fibroin–modified thiol-branched graphene oxide quantum dots (Supplementary Material Fig. S1d) [117]. This method efficiently enables the proportional fluorescence detection of tetracycline in real samples, demonstrating a linear range of 0–90 nM, with notably low detection limits across various samples, such as deionized water, chicken, fish, human serum, and honey (49.69, 47.76, 55.25, 47.90, and 45.78 nM, respectively). The enhanced luminescence properties of the material in liquid media are visible to the naked eye under 365 nm ultraviolet light, allowing the smartphone to detect color changes during sensing and translate them into readable Red-Green-Blue (RGB) data using 365 nm light-emitting diode. The portable sensor boasts a detection limit of 0.125 μM, facilitating swift onsite tetracycline detection.

#### 3.1.3 Surface-enhanced Raman scattering-based detection methods

Surface-enhanced Raman scattering (SERS) is a pivotal tool in the detection and serves as an exceptionally sensitive optical identification methodology [[118], [119], [120]]. One pioneering study involved the creation of silver nanoparticle (AgNP) arrays through template-assisted electrochemical deposition of anodized aluminum oxide (AAO) (Supplementary Material Fig. S1e). In this study, scientists meticulously fine-tuned the periodic nanostructure to increase SERS activity by altering the nanopore diameter and silver NP sputtering time. Consequently, they managed to reduce the gap between silver NPs to less than 10 nm, effectively enabling the detection of tetracycline in milk at impressively low concentrations (1 × 10^−9^ M) [121]. This breakthrough highlights the potential of optimized nanostructures to push the boundaries of sensitive detection methodologies.

#### 3.1.4 Electrochemistry-based detection methods

Electrochemical detection methods are gradually used in tetracycline trace detection [[122], [123], [124], [125]]. Researchers utilized the current values corresponding to the oxidation peak at −1.06 V and the reduction peak at 0.724 V of the electrochemically active metal‒organic frameworks Mo@MOF-808 and NH_2_-UiO-66 as response signals for detecting ultratrace levels of tetracycline. The analysis focused on monitoring changes in the intensities of the currents associated with these specific voltages, providing a sensitive and precise method for tetracycline detection [126]. This technique was utilized for the ultratrace detection of tetracycline (Supplementary Material Fig. S1f). Notably, this sensor demonstrates an extensive linear range for tetracycline (0.1–10000 nM) and an impressively low detection limit of 0.009792 nM. Furthermore, the sensor exhibits superior sensitivity, repeatability, and stability compared to single-signal sensors.

#### 3.1.5 Scope and limitations of detection methods

For the detection of tetracycline, particularly in complex aquatic environments, the sample complexity must be addressed to identify the appropriate detection method. Fluorescence sensors offer high selectivity in detecting antibiotics in aquaculture wastewater, enabling the identification of specific antibiotics. However, fluorescence sensors are prone to photobleaching, which can degrade the sensor signal over time, compromising long-term stability and accuracy. Additionally, the complexity of aquaculture wastewater further impacts the performance of fluorescence sensors, making detection challenging and prone to error. SERS sensors face several challenges in practical applications, including their sensitivity, reproducibility, and range of applicability, particularly in complex aquatic environments, such as aquaculture wastewater. Electrochemical sensors encounter long-term stability issues in practical applications and can be influenced by environmental interference. Furthermore, owing to the widespread use of tetracyclines, there is a need for rapid, onsite, portable monitoring beyond the laboratory setting. This creates new challenges for detection methods. To overcome these limitations, emerging analytical strategies based on alternative mechanisms, such as electronic sensors, intelligent colorimetric sensors, and integrated electrochemical probes, are becoming essential for rapid analysis in diverse aquatic environments. These detection methods have become major focal points of research.

### 3.2 Detection of macrolide antibiotics

There is a lack of effective methods for detecting residues of azithromycin, a prominent macrolide antibiotic widely utilized in animal husbandry and aquaculture for respiratory antibacterial purposes. Innovative approaches predominantly rely on electrochemical principles [[127], [128], [129], [130]], with limited strategies reported.

#### 3.2.1 Electrochemistry-based detection methods

**Fig. 3:**
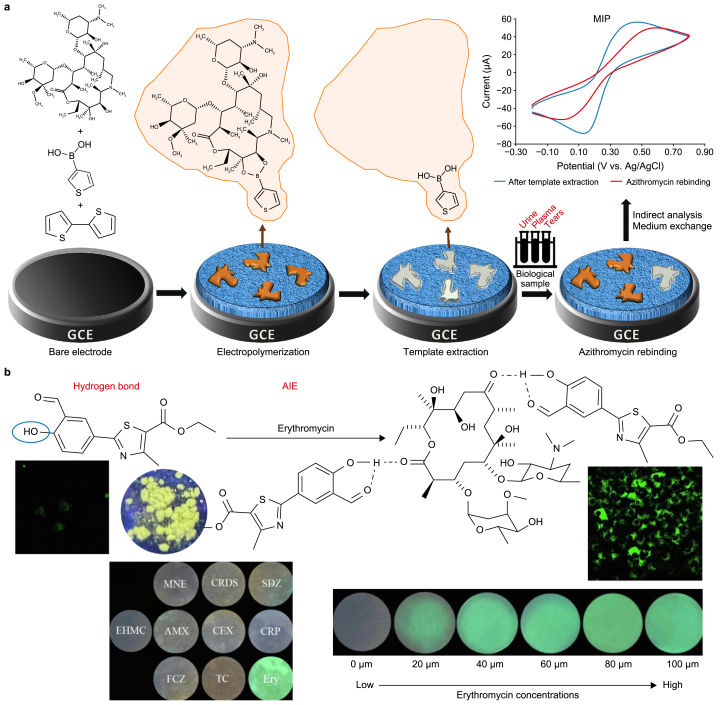
Typical analysis and detection methods for azithromycin and erythromycin. **a**, Electrochemical method. MIP: molecularly imprinted polymer; GCE: glassy carbon electrode. Adapted from Ref. [131]. Copyright 2020, Elsevier. **b**, Fluorescence method. AIE: aggregation-induced emission. Adapted from Ref. [135]. Copyright 2023, Elsevier.

A typical work in 2020 proposed an electrochemical sensor for measuring azithromycin in various complex liquid environments (urine, tears, and plasma) [131]. This method hinges on using electrodeposition to craft molecularly imprinted polymers (MIPs) to form a bionic recognition layer approximately 75 nm thick on a glassy carbon electrode (Fig. 3a). The sensor exhibits a dynamic response range of 13.33–66.67 μM and boasts a remarkable detection limit of 0.85 nM for azithromycin. In addition to its impressive sensitivity, this sensor has demonstrated practical application viability in challenging environments, such as urine, tears, and plasma. Its advantages include a straightforward reusable design, an extended shelf life, and promising prospects for detecting macrolide antibiotics, specifically azithromycin.

#### 3.2.2 Fluorescence-based detection methods

In livestock and aquaculture fields, erythromycin is a common treatment for various bacterial infections. Unfortunately, this antibiotic frequently occurs in water environments [84,[132], [133], [134]]. A detection method was devised to address this issue using a fluorescent probe with aggregation-induced emission characteristics both in a solid state and in water/ether binary solvents [135]. This innovative approach allows for the identification of erythromycin in water. Moreover, a portable test strip developed based on this method enables precise and selective detection and determination of the concentration of erythromycin in diverse and complex settings, including various water sources and living cells (detectable down to a limit of 17.8 nM). Remarkably, the test paper exhibited robust green fluorescence only in the presence of erythromycin among the different antibiotic solutions (Fig. 3b). When various concentrations of erythromycin solutions were applied to the test paper, a distinct amplification of green fluorescence was visually apparent. This observation highlights the promising potential of this fluorescent test paper in evaluating erythromycin contamination.

#### 3.2.3 Scope and limitations of detection methods

Detection methods for macrolide antibiotics with large rings have focused primarily on simple and cost-effective optical and electrochemical sensing strategies. This is mainly due to the high cost of chromatography‒mass spectrometry methods, which require complex equipment. These factors significantly limit the potential application of these methods for the portable and rapid detection of macrolide antibiotics. Therefore, developing new detection methods based on optical and electrochemical techniques holds great promise. Addressing practical needs and applying these methods in complex water environments remain challenging. Developing advanced materials with large surface areas and multiple reaction sites for electrochemical sensors can improve sensitivity and selectivity, but this remains challenging to achieve. Fluorescence sensors face several challenges in practical applications, such as reproducibility and application range. The reproducibility issue requires the introduction of internal standards and the preparation of highly uniform fluorescent materials. Additionally, expanding the application range of fluorescence sensors for the rapid *in situ* detection of macrolide antibiotics in aquaculture wastewater is crucial.

### 3.3 Detection of sulfonamide antibiotics

Detection methods for sulfonamide antibiotics have focused primarily on optical and electrochemical techniques [[136], [137], [138], [139], [140]].

#### 3.3.1 Fluorescence-based quinolone antibiotic detection methods

**Fig. 4:**
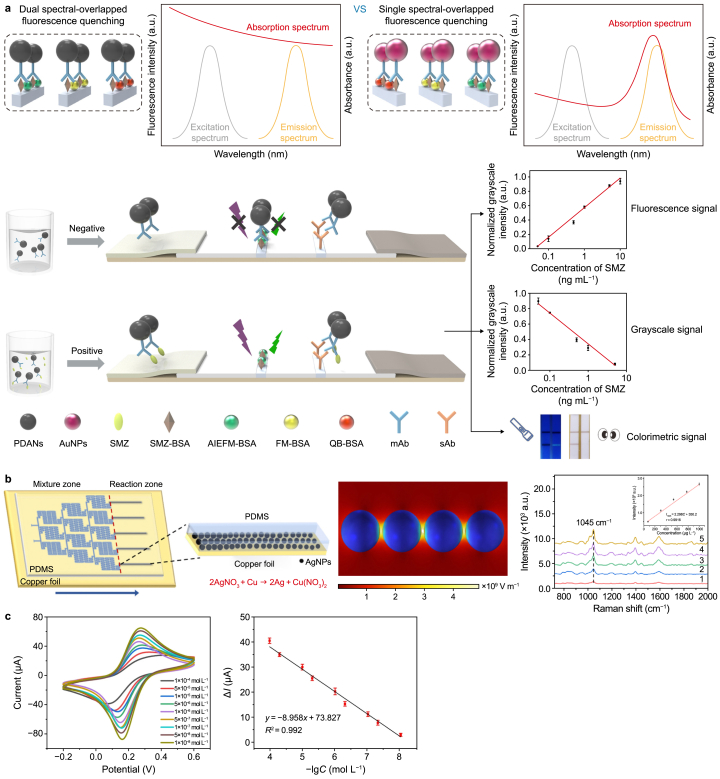
Typical analysis and detection methods for sulfonamide antibiotics. **a**, Fluorescence method. PDANs: polydopamine nanospheres; AuNPs: gold nanoparticles; SMZ: sulfamethazine; BSA: bovine serum albumin; AIEFM: aggregation-induced emission fluorescent microsphere; QB: quantum dot bead; FM: fluorescent microsphere; mAb: monoclonal antibody; sAb: secondary antibody. Adapted from Ref. [137]. Copyright 2022, Elsevier. **b**, Surface-enhanced Raman scattering method. PDMS: polydimethylsiloxane; AgNPs: silver nanoparticles. Adapted from Ref. [147]. Copyright 2024, Royal Society of Chemistry. **c**, Electrochemical method. DMSP: dimethyl sulfoxide; HAc: acetic acid; SDZ: sulfadiazine. Adapted from Ref. [151]. Copyright 2024, Springer.

Fluorescence detection is a highly specific method commonly used to recognize antibiotics [[141], [142], [143]]. A 2022 study proposed a transverse-flow antibiotic assay for the sensitive detection of sulfamethoxazole based on dual-spectral overlapping fluorescence quenching of polydopamine nanospheres (PDANs) due to the internal filtering effect (Fig. 4a) [137]. The device consists of four components: an absorbent pad, a polyvinyl chloride pad, a sample pad, and a nitrocellulose membrane. Compared to traditional quenchers, PDANs can quench three types of fluorescent donors: aggregation-induced emission fluorescent microspheres, excitation and emission light of fluorescent microspheres, and quantum dot beads. This results in a significant increase in fluorescence intensity and a higher signal-to-noise ratio. The detection and visual detection limits of sulfamethoxazole in chicken meat obtained using this method are 0.043 and 0.5 ng mL^−1^, respectively. This method is also applicable for the onsite detection of aquaculture wastewater, demonstrating its practical application potential.

In 2024, Zhang et al. developed a method for detecting sulfamethoxazole based on photoinduced electron transfer between imidazole ligands and copper ions [144]. Surface-modified upconversion nanoparticles bind to Cu^2+^ through electrostatic adsorption, leading to fluorescence quenching. The sensor exhibits excellent linearity over a wide concentration range (0.05–1000 ng mL^−1^), with a detection limit of 0.04 ng mL^−1^. It is highly selective and is unaffected by common substances in aquatic media. These findings indicate that the sensor has great potential for detecting residual sulfamethoxazole in aquatic products.

#### 3.3.2 Surface-enhanced Raman scattering-based quinolone antibiotic detection methods

The SERS technique is a sensitive detection method frequently used to recognize sulfonamide antibiotics [[145], [146], [147], [148]]. Sun et al. designed a multifunctional structure containing gold nanoparticles embedded within a zirconium-based metal‒organic framework [146]. Methods such as analyte enrichment, Raman signal enhancement, and internal standard calibration demonstrated excellent SERS-sensing performance for sulfonamide antibiotics (sulfamethoxazole, sulfamethoxazole, sulfamethoxazole, and sulfaquinoxaline). The extensive π-bond conjugation system of Zr-MOF significantly enhances the concentration of sulfonamide antibiotics through π–π interactions. The embedded antibiotics are vertically adsorbed on the Au surface through coordination interactions between Au and two oxygen atoms or between Au and the terminal amino group. The above four antibiotics were detected in real honey samples at concentrations ranging from 2.8 to 5.2 ng g^−1^ within 10 min.

In the same year, Huang et al. designed a copper/polydimethylsiloxane microfluidic chip (Fig. 4b) [147]. SERS substrates were synthesized through displacement reactions between silver and copper in microfluidic channels, forming a microfluidic SERS nanoparticle (Ag NPs)/Cu chip. Combined with a concentration gradient microchannel network design, this chip can generate a standard curve for a sample with a single injection on a single chip. The linear range for sulfamethoxazole analysis is 75–1000 mg L^−1^, with a detection limit of 28.4 μg L^−1^. Sulfonamide pyrimidine was detected in aquatic products, with recovery rates ranging from 82.7 % to 110 %. The entire testing process for sulfamethoxazole detection can be completed within 20 min.

#### 3.3.3 Electrochemistry-based quinolone antibiotic detection methods

Recently, many detection methods based on electrochemical methods have been proposed [[149], [150], [151], [152], [153]].

One notable study stands out because of the wide linear range (1 × 10^−8^‒1 × 10^−4^ mol L^−1^) and low detection limit (1.30 × 10^−9^ mol L^−1^). The researchers developed a novel molecularly imprinted electrochemical sensor for the specific detection of sulfadiazine in aquaculture feed (Fig. 4c) [151]. Niobium carbide (Nb_2_CT_*x*_), a typical two-dimensional layered nanomaterial, has good conductivity and a unique structure. It was assembled with one-dimensional silver nanowires (AgNWs) to form quasi-3D composite nanomaterials (Nb_2_CT_*x*_/AgNWs) (Supplementary Material Fig. S2). As a spacer material, AgNWs prevent aggregation of Nb_2_CT_*x*_ and the collapse of the Nb_2_CT_*x*_ layer. Simultaneously, the synergistic effect between the nanomaterials creates a rapid electron transfer channel. The Nb_2_CT_*x*_/AgNW composite enhances the electrical signals. This approach has been successfully applied to detect sulfamethoxazole in aquaculture feed.

#### 3.3.4 Scope and limitations of detection methods

In sulfonamide antibiotic detection, emerging strategies have focused primarily on fluorescence, SERS, and electrochemical methods. This is primarily due to the complexity of chromatography‒mass spectrometry methods and the need for expensive equipment. These factors limit the applicability of these methods for portable and rapid sulfonamide antibiotic detection. Emerging detection strategies require developing new materials and interdisciplinary collaboration among fields such as biology, chemistry, and environmental monitoring. Furthermore, these methods should not be confined to laboratory settings but should be adaptable to real-world environments, which present unique challenges for practical implementation. Overall, intelligent visual colorimetric sensing and portable SERS detection devices will likely become promising key approaches for sulfonamide antibiotic detection. These methods are expected to enable the *in situ* detection of sulfonamide antibiotics in aquaculture wastewater.

### 3.4 Detection of quinolone antibiotics

Quinolone antibiotics are commonly used in the breeding industry; thus, their effective detection is crucial. Typical quinolone antibiotics include ofloxacin, ciprofloxacin, and norfloxacin. Detection methods for ofloxacin focus predominantly on optical and electrochemical techniques [[154], [155], [156], [157], [158]].

#### 3.4.1 Fluorescence-based quinolone antibiotic detection methods

In 2022, researchers proposed a multianalyte fluorescent probe based on biocompatible chitosan-capped CdS (CTS-CdS) quantum dots to detect environmentally harmful organic contaminants [154]. A straightforward water chemistry method was employed for the probe to prepare the CTS-CdS quantum dots. The probe demonstrates unique fluorescence properties with high selectivity for various contaminants in wastewater. Specifically, it shows a lower detection limit (0.006 μM) for ofloxacin and exhibits good stability for the trace detection of organic contaminants in real samples.

**Fig. 5:**
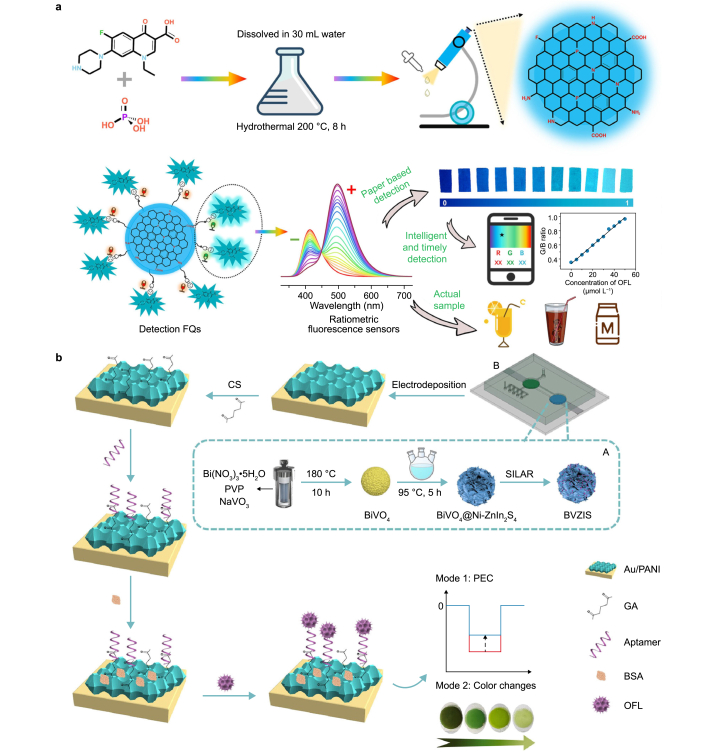
Typical analysis and detection methods for ofloxacin. **a**, Fluorescence/colorimetric method. Adapted from Ref. [159]. Copyright 2023, Elsevier. **b**, Electrochemical method. CS: chitosan; BVZIS: BiVO_4_@Ni-ZnIn_2_S_4_/Bi_2_S_3_; SILAR: successive ionic layer adsorption and reaction; PVP: polyvinyl pyrrolidone; GA: glutaraldehyde; BSA: bovine serum albumin; OFL: ofloxacin. Adapted from Ref. [160]. Copyright 2023, American Chemical Society.

In 2023, a groundbreaking study introduced a novel ratiometric fluorescence detection approach for ofloxacin and its l-isomer levofloxacin (LEV) using tridoped graphene quantum dots (T-GQDs) [159]. This innovative method harnessed norfloxacin and phosphoric acid as doping sources to synthesize T-GQDs in a single step. An intelligent RGB‒time color analysis method was also devised in this study based on fluorescent test paper, enabling the real-time detection of OFL/LEV (Fig. 5a). The fundamental concept revolves around the electrostatic bond between T-GQDs and OFL/LEV, which induces an intermolecular electron transfer effect. This transfer reduces fluorescence intensity as electrons move from the T-GQDs to OFL/LEV. The sensor achieves impressive detection limits of 46 and 67 nM for the two antibiotics. Moreover, it can detect these substances in complex water environments, such as juice, carbonated drinks, and milk. In the same year, a rapid and energy-efficient two-step synthesis method was developed to produce self-passivated fluorescent water-soluble CDs (wsCDs) from sustainable microcrystalline cellulose materials [156]. The aqueous solution of wsCD exhibits blue emission under ultraviolet light, with a fluorescence quantum yield of approximately 6 %. Additionally, wsCDs were employed for the specific detection of ofloxacin, achieving a detection limit of approximately 0.025 ppm. Antibacterial experiments with wsCDs indicated no toxic effects at a concentration of 1 mg mL^−1^, supporting their biocompatibility.

#### 3.4.2 Electrochemistry-based quinolone antibiotic detection methods

Along a different line of research, scientists have introduced an electrochemical analysis method for detecting ofloxacin [160]. This method involves a dual-mode microfluidic analysis device combining photoelectrochemical sensing and electrochromic visual analysis to detect ofloxacin. A stable electron supply is provided in this strategy by designing a dual direct z-scheme BiVO_4_@Ni-ZnIn_2_S_4_/Bi_2_S_3_ heterojunction as a photoanode (Fig. 5b). The dual z-scheme structure of the composite expedites electron migration. A polyaniline-modified electrochromic material, Au/PANI, was subsequently electrodeposited on the photocathode to affix the aptamer and enable visual reading. The Au/PANI material directly accepts electrons from the photoanode, causing a rapid decrease in polyaniline and a visible color change from blue to green. This method shows remarkable OFL electrochemical and colorimetric (RGB-green) detection limits of 18 and 30 fg mL^−1^, respectively, enabling the sensitive detection of OFL in milk and river water.

#### 3.4.3 Quantum dot fluorescence-based quinolone antibiotic detection methods

In recent years, there has been a surge in proposals of various innovative methods for detecting ciprofloxacin, marking a significant advancement in sensing technologies [[161], [162], [163], [164], [165], [166], [167], [168]].

**Fig. 6:**
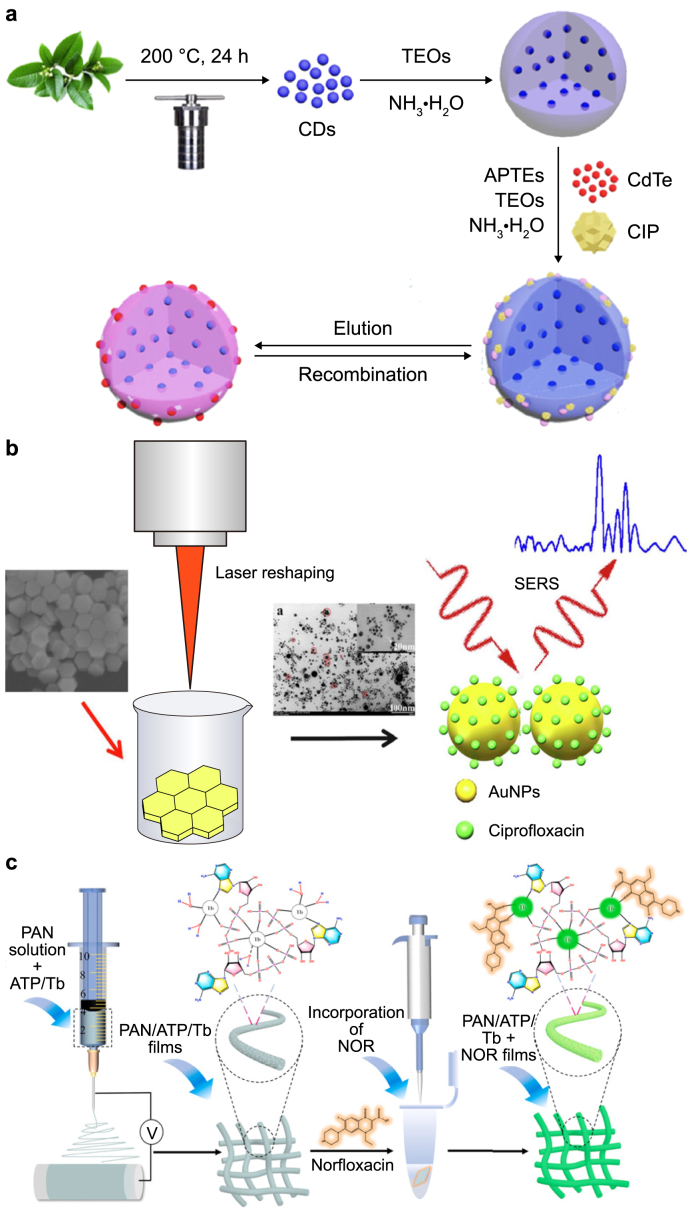
Typical analysis and detection methods for ciprofloxacin and norfloxacin. **a**, Fluorescence method. CDs: carbon dots; TEOs: tetraethoxysilane; APTEs: 3-aminopropyltriethoxysilane; CIP: ciprofloxacin. Adapted from Ref. [167]. Copyright 2019, Elsevier. **b**, Surface-enhanced Raman scattering (SERS) method. AuNPs: gold nanoparticle. Adapted from Ref. [169]. Copyright 2022, Elsevier. **c**, Fluorescence method. PAN: polyacrylonitrile; ATP: adenosine triphosphate; NOR: norfloxacin. Adapted from Ref. [179]. Copyright 2023, Elsevier.

Researchers have presented a pioneering approach to ciprofloxacin sensing by developing a multifunctional composite material composed of carbon dots and CdTe quantum dots [167]. This innovative composite material was created using osmanthus leaves as a carbon source and polyethylenimine as a nitrogen source to generate highly fluorescent CDs through a hydrothermal method (Fig. 6a). The resulting blue CDs were combined with red CdTe quantum dots to construct MIPs@CdTe/CDs@SiO_2_. The selectivity for and sensitivity to ciprofloxacin were evaluated using TiO_2_/CDs/CdTe quantum dots as photocatalysts for ciprofloxacin degradation. The strategy exhibits a remarkable detection limit for ciprofloxacin of 0.0127 nM and demonstrates a linear testing range of 0–60 nM. It was successfully applied to monitor ciprofloxacin levels in human urine.

#### 3.4.4 Surface-enhanced Raman scattering-based quinolone antibiotic detection methods

SERS is frequently employed as a prominent optical detection method for quinolone antibiotic detection. Researchers manipulated femtosecond and nanosecond lasers to shape hexagonal gold sheets into particles of various sizes and forms and then utilized them for the label-free SERS detection of ciprofloxacin (Fig. 6b) [169]. By reshaping gold nanoparticles into spherical–hexagonal structures with a diameter of 120 nm and spherical–triangular combinations with a diameter of 6 nm via laser action, this method achieves a remarkable LOD of 7.5 × 10^−8^ M for ciprofloxacin in milk. It leverages the ultrafast laser reshaping of gold nanoparticles, paving the way for the straightforward and portable detection of contaminants.

Semiconductors have increasingly been used to prepare SERS substrates [[170], [171], [172]]. In 2023, Jiang et al. successfully synthesized multifunctional materials using a layer-by-layer method. Fe_3_O_4_@mTiO_2_@Ag core‒shell nanoparticles enabled the *in situ* detection of quinolone antibiotics in aquaculture wastewater from water plants [173]. The results revealed that the minimum detectable concentrations of the investigated antibiotics were 1 × 10^−9^ M (ciprofloxacin, enrofloxacin, and norfloxacin) and 1 × 10^−8^ M (difloxacin hydrochloride), and the detection performance was enhanced by Fe_3_O_4_@mTiO_2_@Ag NPs. Furthermore, a strong quantitative relationship was observed between the antibiotic concentration and the SERS peak intensity within a specific detection range. The spiked assay results for actual aquaculture water samples revealed that the recoveries for six antibiotics ranged from 82.9 % to 113.5 %, with relative standard deviations ranging from 1.71 % to 7.24 %. This approach provides a multifunctional solution for the low-concentration detection and effective degradation of antibiotics in aquaculture water.

#### 3.4.5 Method for detecting quinolone antibiotics based on SERS microfluidic channels

The combination of microfluidic and SERS detection strategies is becoming increasingly popular [[174], [175], [176], [177]]. In a distinct approach, researchers integrated SERS substrates onto the surfaces of optical fiber cores within microfluidic channels to detect ciprofloxacin and norfloxacin residues in water [178]. This innovative strategy entails using self-assembled Ag particles as the SERS substrate and constructing a microfluidic sensing platform through hollow-core optical fibers (Supplementary Material Fig. S3). The experimental results revealed LODs for ciprofloxacin (0.1 nM) and norfloxacin (0.01 nM) substantially below the maximum residue limit specified by the European Union (3.01 × 10^−7^ M). This solution offers a novel approach for the label-free microfluidic detection of antibiotic residues within optical fibers and holds promise for applications in the realm of environmental antibiotic water pollution detection.

#### 3.4.6 Electrospinning-based quinolone antibiotic detection methods

A different approach was developed using electrospinning technology to combine polyacrylonitrile fibers with adenosine triphosphate (ATP)-rare earth metal Tb^3+^ complexes (ATP/Tb) for the onsite detection of norfloxacin in a thin-film sensor (Fig. 6c) [179]. The main operational mechanism involves the gradual enhancement of the bright green fluorescence produced by the system in the presence of norfloxacin. This sensor exhibits a strong linear response to norfloxacin within a wide concentration range of 0.04–30 μM, with a detection limit of 16 nM. When coupled with a smartphone color recognition application, this thin-film sensing strategy facilitates onsite norfloxacin testing. The development of this strategy, coupled with intelligent portable identification, holds considerable significance for rapid detection in water environments.

#### 3.4.7 Scope and limitations of detection methods

Quinolone antibiotic detection strategies focus predominantly on electrochemical and fluorescence methods. The success of these strategies relies heavily on integrating novel materials and necessitates interdisciplinary collaboration across the fields of biology, chemistry, and environmental monitoring, creating new challenges for the research community. The main limitations of electrochemical sensors are their poor repeatability, stability, and portability. Fluorescence sensors are prone to signal degradation over time due to photobleaching and face challenges in preparing complex materials. Moreover, these methods are highly susceptible to the complex composition of aquaculture wastewater. Additionally, these methods should be extended beyond laboratory settings and applied to real-world environments, which presents unique challenges for practical implementation. Overall, intelligent visual colorimetric sensing, electrochemical trace detection, and similar strategies hold promise as crucial approaches for feasible quinolone antibiotic detection. However, these methods must be capable of *in situ* detection in real-world environments.

### 3.5 Detection of β-lactam antibiotics

β-lactam antibiotics, predominantly penicillin and its derivatives, such as amoxicillin, are extensively used in animal husbandry with notable irregularities. Consequently, the precise detection of β-lactam antibiotics is highly important [[180], [181], [182], [183], [184], [185]].

#### 3.5.1 Surface-enhanced Raman scattering-based β-lactam antibiotic detection methods

**Fig. 7:**
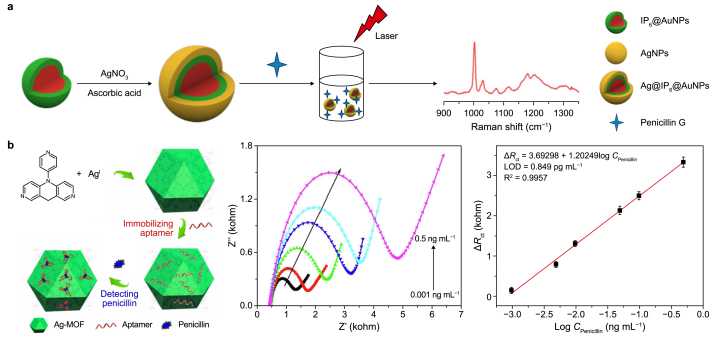
Typical analysis and detection methods for β-lactams and erythromycin. **a**, Surface-enhanced Raman scattering method. Adapted from Ref. [184]. Copyright 2021, Elsevier. **b**, Electrochemical method. MOF: metal–organic framework; LOD: Limit of detection. Adapted from Ref. [188]. Copyright 2020, Elsevier.

A conventional approach involves the creation of a SERS substrate known as Ag@IP_6_@AuNPs (Fig. 7a) [184]. This substrate was achieved by enveloping gold nanoparticles protected by inositol hexaphosphate (IP_6_) within a silver shell. The efficacy of this substrate is evident, as it was successfully used to identify trace amounts of penicillin residues in milk, demonstrating a linear quantitative detection range between 10^−5^ and 10^−11^ M, with a detection limit of 10^−3^ nM. Meeting the sensitivity requirements stipulated by the European Union's maximum residue limit for penicillin in dairy products (1.2 × 10^−8^ M), this detection system holds substantial promise for practical application.

#### 3.5.2 Electrochemistry-based β-lactam antibiotic detection methods

Electrochemical sensing strategies also play a pivotal role in penicillin detection [185,186]. For example, a carbazole-based porous organic polymer (POP) is synthesized via the Friedel−Crafts coupling reaction between 2,4,6-tri(9H-carbazol-9-yl)-1,3,5-triazine and 2,4,6-tris(bromomethyl)mesitylene. The synthesized POP can immobilize aptamers to create an electrochemical aptasensor that detects penicillin with exceptional sensitivity, high selectivity, excellent reproducibility, and remarkable stability. Furthermore, the POP-based electrochemical aptasensor can quantitatively detect ultratrace levels of penicillin (0.001 ng mL^−1^) in real-world samples [187].

In another typical work, researchers engineered a biosensor based on Ag(I)-MOF materials by assembling Ag(I) salts of tripyridin-4-ylamine with distinct anions (SiF_6_^2−^ versus CH_3_SO_3_^−^) (Fig. 7b) [188]. This biosensor exhibits exceptional selectivity for penicillin under interference from other antibiotics and boasts an impressive minimum detection limit of 0.849 pg mL^−1^, effectively monitoring penicillin content in milk samples.

#### 3.5.3 Fluorescence-based β-lactam antibiotic detection methods

The current phase of innovative amoxicillin detection strategies predominantly emphasizes [26,[189], [190], [191]] using electrochemical and fluorescence colorimetric methods. A notable example of amoxicillin detection via an electrochemical approach has previously been highlighted [87,104]. In addition to the aforementioned electrochemical detection method, in a different study, fluorescent apparatus employing specially synthesized green safety materials, i.e., red carbon dots (RCDs) and blue carbon dots (BCDs), was engineered (Supplementary Material Fig. S4). This nanoprobe was designed for amoxicillin detection [192]. Fundamentally, the premise of this research lies in the occurrence of a reaction that induces hydrogen bonding in the presence of amoxicillin. This reaction notably amplifies the blue fluorescence intensity of the BCDs, resulting in a significant color change from red to blue. Furthermore, for practical applications, the tailored fluorescent handheld probe, when integrated with smart devices, enables the direct and quantitative detection of amoxicillin in real samples. This device boasts a fast detection time and a notably low detection limit (2.39 nM). The efficacy of this approach suggests promise for application in the precise trace detection of amoxicillin.

#### 3.5.4 Scope and limitations of detection methods

Currently, most β-lactam antibiotic detection methods are confined to laboratory settings, and their practical deployment is limited by the complex preparation required for sensing materials. Additionally, the sensors show limited adaptability to various environmental factors, such as pH, temperature, and humidity. Therefore, a key challenge moving forward is to enable the application of β-lactam antibiotic sensors in real-world environments. For example, fluorescence combined with smart devices, such as smartphones, as discussed in Section 3.5.3, represents an effective onsite wastewater detection strategy. However, issues related to the stability and rapid analysis of optical metrology and software remain, requiring advanced software support and adding to operational challenges.

## 4 Challenges and potential solutions

Table 3 provides a comprehensive overview of the detection strategies utilized for various prominent antibiotics and their corresponding detection performance metrics. These methods serve as valuable reference points in the dynamic landscape of antibiotic testing. In the future, efforts should focus on streamlining sample processing, improving the portability of analytical equipment, reducing usage costs, and ensuring the clarity and intuitiveness of test results. These coordinated efforts will significantly contribute to advancing the effectiveness and accessibility of antibiotic testing methodologies.

**Table 3:** Detection performance of various representative antibiotic detection strategies.

Antibiotic	Detection strategy	Examination range	Detection limit	Reference
Tetracycline	Fluorescence	0–160 μM	1.73 nM	[113]
Ofloxacin	Colorimetric	0–90 nM	4.78 × 10^−1^ nM	[117]
Ciprofloxacin	SERS	0–10^4^ nM	1 nM	[119]
Norfloxacin	Electrochemistry	0–30 μM	9.792 × 10^−3^ nM	[126]
Azithromycin	Fluorescence	0.1–10^5^ pg mL^−1^	46 nM	[159]
Erythromycin	Electrochemistry	0–60 nM	18 fg mL^−1^	[160]
Penicillin	Fluorescence	0.1–10^6^ nM	0.0127 nM	[167]
Amoxicillin	SERS	0.04–30 μM	0.1 nM	[178]
	Fluorescence	0.01–10^6^ nM	16 nM	[166]
	SERS	13.33‒6 × 10^4^ μM	0.01 nM	[178]
	Electrochemistry	0–100 μM	0.85 nM	[131]
	Fluorescence	10^−5^‒10^−11^ M	17.8 nM	[135]
	SERS	1.43–429.12 μM	10^−3^ nM	[184]
	Electrochemistry	0–6 μM	0.849 pg mL^−1^	[188]
	Fluorescence	0–100 nM	825 nM	[26]
	Colorimetric		2.39 nM	[192]
	Electrochemistry		15 nM	[104]

## 5 Summary and future prospects

In recent decades, as the quality of industrial and agricultural production has improved, there has been a growing emphasis on material needs for a better quality of life and for protecting the environment, which are crucial for survival. This emphasis has led to a significant increase in demand for the detection of residual antibiotics in the environment since they are emerging contaminants in various types of wastewater. Many detection and analysis strategies have been reported to effectively detect concentrations of emerging antibiotic contaminants in the natural environment. Generally, gas chromatography‒mass spectrometry, a traditional method for antibiotic detection, possesses characteristics such as high precision, sensitivity, and good selectivity. However, this method has several disadvantages, including expensive instrumentation, lengthy pretreatment times, and the need for specialized operators, which greatly limits its practical application. Consequently, numerous emerging detection methods have been introduced, such as microbiological analysis, optical methods, and electrochemical sensing. This paper explored the primary sources and types of residual antibiotics in wastewater from livestock and aquaculture, briefly reviewed various detection methods and their principles, outlined several emerging approaches for detecting typical antibiotics, and summarized the detection performance of each method, highlighting their excellent detection limits.

**Fig. 8:**
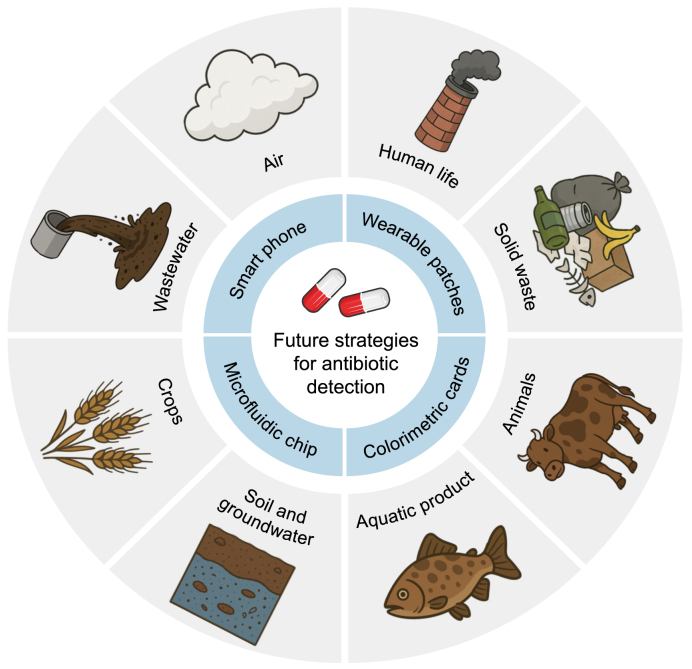
Potential strategies for detecting antibiotics in various complex environments.

To meet the market demand for efficient and cost-effective strategies for detecting antibiotics in complex water environments, detection methods and sensor structures should be tailored to specific needs and detection conditions. Despite numerous reported methods for detecting antibiotics in complex environments, these strategies still face limitations and shortcomings in widespread application. Significant progress has been made in microbial methods for antibiotic detection. Microbial sensing strategies offer high specificity for detecting trace levels of antibiotics. However, practical applications pose challenges adapting to sensing platforms and selecting appropriate biomolecules. Ensuring compatibility between materials and biomolecules and establishing stable and effective connections between biomolecules and the target substance are crucial considerations. These issues are key challenges for the future of microbial detection strategies. Compared to traditional analytical approaches, optical antibiotic detection methods (e.g., fluorescence, colorimetric, and SERS) offer speed, intuitiveness, and relatively low detection limits. Moreover, when integrated with intelligent platforms, these methods demonstrate the advantage of rapid onsite monitoring. However, the preparation of fluorescent probes, SERS substrates, and various new materials, as well as the potential introduction of new pollution sources into aquatic environments through these probes, poses practical challenges that must be addressed. Electrochemical sensing methods offer ultralow detection limits and high sensitivity, making them promising for trace detection applications. However, these methods encounter similar challenges regarding probe preparation, the outdoor stability of electrochemical workstations, and portability. In the future (Fig. 8), integrating sensors with the Internet of Things (IoT) or artificial intelligence technologies will enable remote data acquisition, online analysis, and cloud database upload across a broader range of fields. This integration can facilitate sampling and testing at remote locations while efficiently organizing data. For example, combining electrochemical and optical sensors with IoT technology can continuously monitor multiple points in aquaculture wastewater, providing rapid responses to discharging pollutants such as antibiotics. Additionally, the miniaturization and portability of devices, such as portable Raman sensing units, visualized fluorescent test strips, and smartphone image recognition systems, can support rapid onsite detection, ideal for daily inspections with lower precision requirements. These devices, in turn, leverage the specificity of advanced environmentally sensitive materials, offering accurate compositional analyses under various water conditions.

In summary, detecting antibiotics in complex environments via emerging methods involves innovations in material preparation, platform integration, and practical device applications. To address the challenges various detection strategies face, future efforts should prioritize developing stable, highly accurate, portable, cost-effective, and environmentally friendly sensors, thus enabling *in situ* detection in complex aquatic environments. In the future, portable *in situ* detection utilizing smartphones and wearable chips is expected to have a wide range of applications. With advancements in device fabrication technology and updates in integration strategies, we believe that antibiotic detection methods based on new materials and platforms will revolutionize achievements in biology, the environment, health care, and energy.

## CRediT authorship contribution statement

**Xinyu Chang:** Writing – review & editing, Writing – original draft, Visualization, Validation, Software, Resources, Methodology, Investigation, Formal analysis, Data curation, Conceptualization. **Junchi Cui:** Methodology, Formal analysis, Conceptualization. **Guihua Wang:** Investigation, Conceptualization, Resources. **Shujuan Meng:** Software, Resources, Formal analysis, Methodology. **Lingling Chen:** Supervision, Conceptualization, Funding acquisition, Project administration, Writing – review & editing. **Meng Zhang:** Project administration, Funding acquisition, Conceptualization, Supervision, Writing – review & editing.

## Declaration of competing interest

The authors declare that they have no known competing financial interests or personal relationships that could have appeared to influence the work reported in this paper.
