# Supplementary material for: Emerging technologies for detecting antibiotics in aquaculture wastewater: A critical review
Source: Environ Sci Ecotechnol. 2025 May 17;25:100572. doi: 10.1016/j.ese.2025.100572 (PMC12149589; doi:10.1016/j.ese.2025.100572)
Supplement: Multimedia component 1 [file mmc1.docx]

Supplementary Materials

**Detection of antibiotics in water environments: Innovative strategies based on new methods**

Xinyu Chang^a^, Junchi Cui^b^, Guihua Wang^a^, Shujuan Meng^c^, Lingling Chen^d,^ *, Meng Zhang^a,^ *

^a^School of Electronic and Information Engineering, Beihang University, Beijing, 100191, China

^b^School of Environment, Beijing Normal University, Beijing, 100875, China

^c^School of Materials Science and Engineering, Beihang University, Beijing 100191, China

^d^College of Health Science and Environmental Engineering, Shenzhen Technology University, Shenzhen, 518118, China

Phone: 010-82339480

Email:^*^mengzhang10@buaa.edu.cn*; chenlingling@sztu.edu.cn

**Fig. S1.** Typical analysis and detection methods for tetracycline antibiotics. **a**, Fluorescence method. (Carbon dots, CDs. Reduced state carbon dots, r-CDs. Tetracycline, TC) Reprint/Adapted from Ref. [1]. Copyright 2021, Elsevier. **b**, Fluorescence method (Tetracycline: TC). Reprint/Adapted from Ref. [2]. Copyright 2022, American Chemical Society. **c**, Fluorescence method (Tetracycline, TET. Inner filter effect, IFE. Fluorescence resonance energy transfer: FRET. Photo-induced electron transfer, PET). Reprint/Adapted from Ref. [3]. Copyright 2022, Elsevier. **d**, Colorimetric method. Reprint/Adapted from Ref. [4]. Copyright 2023, American Chemical Society. **e**, SERS method. Reprint/Adapted from Ref. [5]. Copyright 2020, American Chemical Society. **f**, Electrochemical method (Tetracycline, TET). Reprint/Adapted from Ref. [6]. Copyright 2023, American Chemical Society.


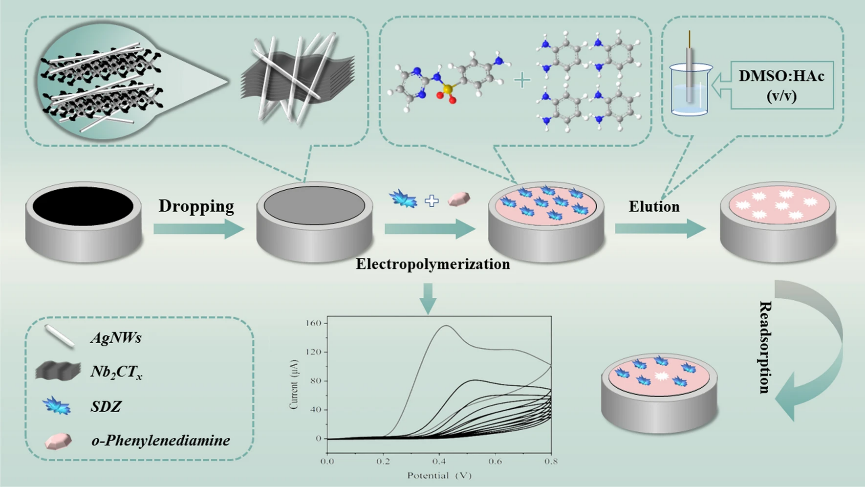


**Fig. S2.** Typical analysis and detection methods for ofloxacin: Electrochemical method (Dimethyl sulfoxide, DMSO. Acetic acid, HAc. Sulfadiazine, SDZ). Adapted from Ref. [7]. Copyright 2024, Springer Viennay.

**
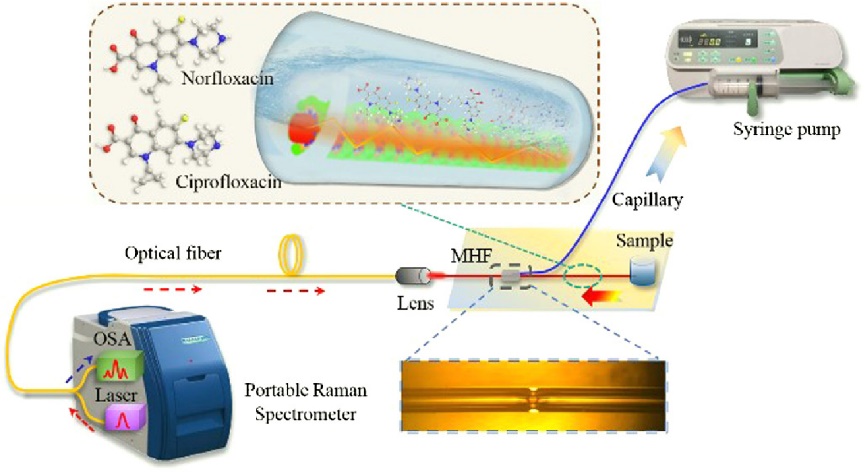
**

**Fig. S3.** Typical analysis and detection methods for ciprofloxacin and norfloxacin: SERS method (Micro-hollow optical fiber, MHF). Adapted from Ref. [8]. Copyright 2021, Optical Society of America.


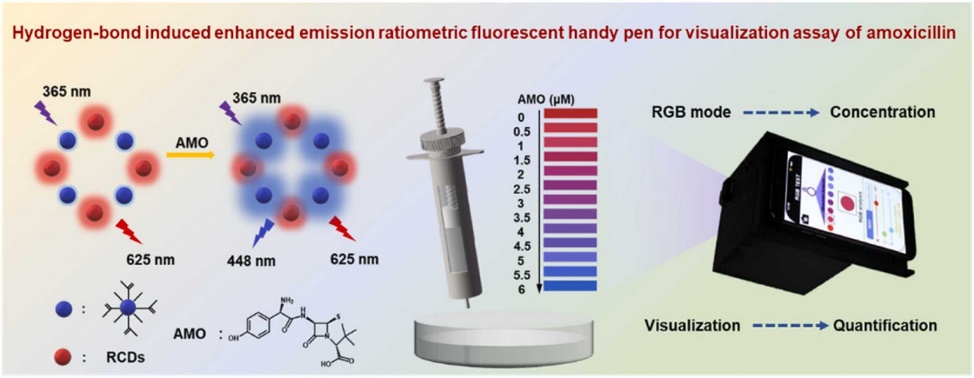


**Fig. S4.** Typical analysis and detection methods for β-lactams and erythromycin: Fluorescence method (Amoxicillin, AMO. Red carbon dots, RCDs). Adapted from Ref. [9]. Copyright 2022, Elsevier.

**References**

1. Q. Fu, C.C. Long, L.F. Qin, Z.X. Jiang, T.P. Qing, P. Zhang, B. Feng, Fluorescent and colorimetric dual-mode detection of tetracycline in wastewater based on heteroatoms-doped reduced state carbon dots, Environ. Pollut. 283 (2021) 117109.
2. M. Bras, J. Zanoni, B.P. Falcao, J.P. Leitao, F.M. Costa, T. Monteiro, et al., Label-Free Nanoscale ZnO Tetrapod-Based Transducers for Tetracycline Detection, ACS Appl. Nano Mater. 5 (2022) 1232‒1243.
3. W. Xin, L.W. Li, J. Hong, Z.S. Hui, Q.Z. Wang, X.Y. Sun, W. Li, Highly selective and sensitive fluorescence detection of tetracyclines based on novel tungsten oxide quantum dots, Food Chem. 374 (2022) 131774.
4. K.J. Goswami, N. Sen Sarma, "Click" Reaction-Mediated Silk Fibroin-Functionalized Thiol-Branched Graphene Oxide Quantum Dots for Smart Sensing of Tetracycline, ACS Omega 8 (2023) 21914‒21928.
5. M. Muhammad, B. Yan, G.H. Yao, K.L. Chao, C.H. Zhu, Q. Huang, Surface-Enhanced Raman Spectroscopy for Trace Detection of Tetracycline and Dicyandiamide in Milk Using Transparent Substrate of Ag Nanoparticle Arrays, ACS Appl. Nano Mater. 3 (2020) 7066‒7075.
6. N.N. Liang, X.T. Hu, X.A. Zhang, W.T. Li, Z. Guo, X.W. Huang, et al., Ratiometric Sensing for Ultratrace Tetracycline Using Electrochemically Active Metal-Organic Frameworks as Response Signals, J. Agric. Food. Chem. 71 (2023) 7584‒7592.
7. Y.F. Wang, J.W. He, J.Wu, W. Hao, L. Cai, H.Y. Wang, G.Z. Fang, S. Wang, A novel molecularly imprinted electrochemical sensor based on quasi-three-dimensional nanomaterials Nb_2_CTx/AgNWs for specific detection of sulfadiazine, Microchim. Acta 191 (2024) 720.
8. P.P. Teng, D.H. Gao, X.H. Yang, M. Luo, D.P. Kong, S. Gao, et al., In situ SERS detection of quinolone antibiotic residues in a water environment based on optofluidic in-fiber integrated Ag nanoparticles, Appl. Opt. 60 (2021) 6659‒6664.
9. L.F. Li, L. Yang, D. Lin, S.H. Xu, C.S. Mei, S.M. Yu, C.L. Jiang, Hydrogen-bond induced enhanced emission ratiometric fluorescent handy needle for visualization assay of amoxicillin by smartphone sensing platform, J. Hazard. Mater. 444 (2022) 130403.
